# Knowledge/attitude/practices of HPV & cervical cancer, willingness to participate in vaccine trial in preparation for HIV & HPV vaccine trials in Mali

**DOI:** 10.1186/1742-4690-9-S2-O27

**Published:** 2012-09-13

**Authors:** D Poole, K Tracy, L Levitz, S Yekta, E Kossow, T Huang, M Rochas, K Sangare, K Tounkara, B Aboubacar, O Koita, F Siby Diallo, S Sow, I Téguété, A Dolo, F Bougoudogo, M Lurie, AS De Groot

**Affiliations:** 1 Brown University, Providence, RI, USA; 2 University of Maryland, College Park, MD, USA; 3 GAIA Vaccine Foundation, Providence, RI, USA; 4 University of Bamako, Bamako, Mali; 5 Fondation GAIA Mali, Bamako, Mali; 6 Direction Regionale de la Sante, Bamako, Mali; 7 Center for Vaccine Development, Bamako, Mali; 8 Hopital Gabriel Touré, Bamako, Mali; 9 Institut National pour la Santé Publique, Bamako, Mali; 10 GAIA Vaccine Foundation, University of Rhode Island, Providence, RI, USA

## Background

The GAIA Vaccine Foundation (GAIA VF) has been collaborating with the Malian regional DOH, local HIV clinicians, and scientists in Bamako to prepare a site for Phase I-III HIV vaccine trials. We recently performed two studies to evaluate HIV and HPV knowledge and willingness to participate (WTP) in an HIV or HPV vaccination trial.

## Methods

Knowledge, Attitudes, and Practices (KAP) studies were performed in 2008 and 2011 to assess KAP related to HIV, HIV transmission, HIV prevention, HPV, cervical cancer, and WTP in vaccine trials. The 2008 KAP study examined HIV KAP and WTP (399 subjects), while the 2011 pilot study examined HPV KAP and WTP for 51 subjects in the same region of Bamako. Results from a more extensive HPV KAP (300 participants) are pending.

## Results

HIV knowledge was high: over 73% of participants in the 2008 study were knowledgeable about modes of HIV transmission. 78% said they would participate in an HIV vaccine trial, 65% in a malaria vaccine trial, and 61% in a tuberculosis vaccine trial. In contrast, in 2011, less than 1% of individuals had heard of HPV. Yet 98% of participants were WTP in an HPV vaccine trial with the aim of obtaining approval of the vaccine in Mali.

## Conclusion

WTP in vaccine trials is high among participants in these West African surveys. In previous African KAP and WTP studies, WTP ranged from 20% to 77% (average 47%). Even though participants were highly willing to participate in an HPV vaccine trial, levels of knowledge were very low. There is a significant need for expanded public education about the link between viruses and infection in West Africa. This study demonstrates challenges in implementing ethical clinical trials and highlights the need for a significant investment in health education if truly informed consent is to be obtained.

